# APJ expression is lost in isolated embryonic coronary endothelial cell culture *in vitro*

**DOI:** 10.17912/micropub.biology.000847

**Published:** 2023-08-11

**Authors:** Bryce Kuschel, Bikram Sharma

**Affiliations:** 1 Department of Biology, Ball State University, Muncie, Indiana, United States

## Abstract

Coronary artery disease is one of the leading causes of death worldwide, and yet we lack the appropriate therapeutic treatments for it. Investigation into the mechanisms of coronary vessel development can provide insights into potential therapies to repair and regenerate damaged coronary arteries. Our previous study in the mouse embryo have implicated APJ, a G-protein coupled receptor that is expressed by coronary endothelial cells *in vivo,* to be an important regulator of coronary vessel development. In this study, we report an unexpected finding that the isolated embryonic coronary endothelial cells lose APJ expression in culture *in vitro.*

**Figure 1. Isolated embryonic coronary endothelial cells in culture lose Apj expression but retain VegfR2 expression:**
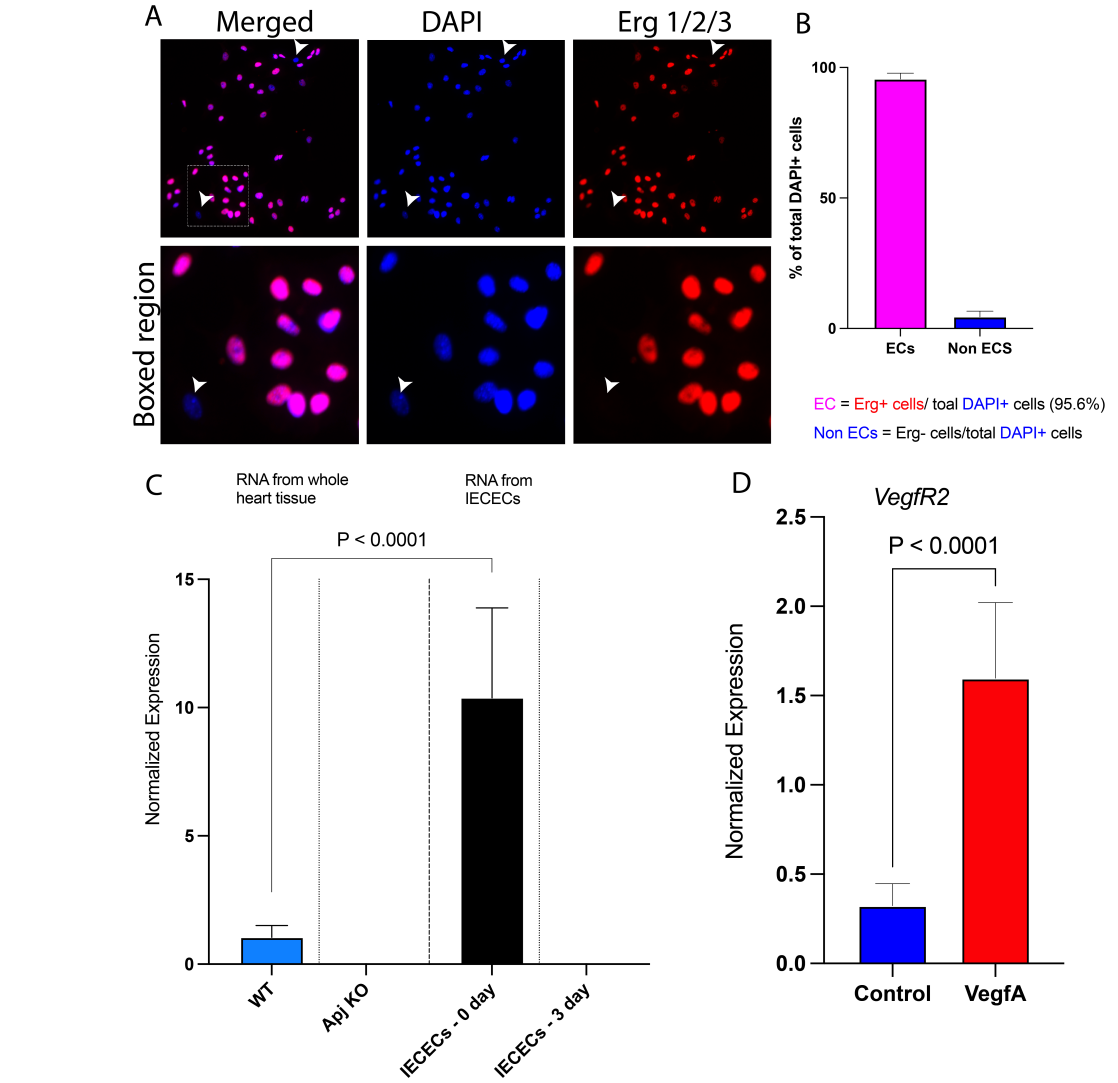
**A)** Isolated coronary endothelial cells in culture stained with Erg 1/2/3 (red) to visualize nuclei of endothelial cells and co-stained with DAPI (blue) to visualize all cell nuclei. Arrowheads depict non-endothelial cells (DAPI ^+^ /Erg 1/2/3 ^-^ ) in the isolated cell population. **B)** Bar graph represents the quantification of endothelial cells (ECs) and Non-ECs percentages in isolated cell culture. **C)** A bar graph representing qRT-PCR data showing expression of *Apj* in the whole heart samples (WT, wildtype; Apj KO, Apj germline Knockout) and the 0-day or 3-day culture of isolated embryonic coronary endothelial cells (IECECs). **D)** Bar graph represents the normalized expression of *VegfR2* in the 3-day culture of isolated embryonic coronary endothelial cells. Data is represented as Mean ± SD. Significance is reported at p < 0.05 (unpaired *t* -test with Welch’s correction).

## Description

Coronary vessels in the mouse embryonic heart develop through angiogenesis from multiple endothelial progenitor pathways (Red-Horse et al., 2010; Wu et al., 2010; Katz et al., 2012; Tian et al., 2013; Chen et al., 2014) . APJ, a G-protein coupled receptor, is implicated in the regulation of coronary angiogenesis, a process by which coronary vessels are formed from its pre-existing progenitors, during embryonic mouse heart development (Sharma et al., 2017; Kang et al., 2013) . In these studies, authors show that coronary angiogenesis is significantly stunted in *Apj* knockout hearts compared to wild-type, suggesting that APJ plays an important role in the regulation of coronary angiogenesis. APJ is activated by two ligands, ELABELA (Sharma et al., 2017; Chng et al., 2013) and APELIN (Kang et al., 2013) , which are expressed by epicardial cells (Sharma et al., 2017) and endothelial cells (Red-Horse et al., 2010) of the developing mouse heart, respectively. During coronary angiogenesis, APELIN + endothelial cells sprout into the subepicardial space where ELABELA is present (Red-Horse et al., 2010; Tian et al., 2013) . Therefore, it is hypothesized that APJ regulates coronary angiogenesis through its dual ligands. However, it is unclear how APJ regulate coronary angiogenesis from its two ligands, ELABELA and APELIN. One of the challenges of such study is the lack of an embryonic coronary endothelial cell line or endothelial cell line with faithful APJ expression to conduct *in vitro* studies. To circumvent this limitation, we set out to isolate primary coronary endothelial cells from embryonic mouse hearts at e14.5, an embryonic stage of active coronary angiogenesis.

We performed primary coronary endothelial cell isolation using the Miltenyi Biotec kit as described in the methods section below. We wanted to assess whether the isolated cell population is highly enriched with endothelial cells as intended. To determine the enrichment of endothelial cells in the isolated pool, we immunostained the isolated cells following a 24-hour culture with endothelial cell-specific antibody anti-Erg1/2/3 to visualize endothelial cell nuclei ( Figure 1A, red ) and co-stained with DAPI ( Figure 1A, blue ). We then analyzed the percentage of endothelial cells (Erg 1/2/3 + / DAPI + cells) versus non-endothelial cells (ERG 1/2/3 - / DAPI + cells). Among the 6,784 total DAPI+ cells that were counted, 6,379 were ERG 1/2/3 + (red- and blue-double stained, purple cells in merged image), whereas 405 were Erg 1/2/3– (blue stain only, indicated by arrowhead) cells in culture. Therefore, our cell isolation yielded a highly enriched endothelial cell population (94%) compared to a low yield of non-endothelial cells (6%) ( Figure 1B ).

Next, we wanted to verify whether the isolated embryonic endothelial cells faithfully expressed APJ when subjected to culture. To analyze this, we performed qRT-PCR on RNA obtained from isolated cells. qRT-PCR analysis confirmed *Apj* expression in freshly isolated non-cultured (0-day culture) isolated embryonic coronary endothelial cells (IECECs), since the RNA from IECECs (n = 4) showed substantially higher *Apj* expression compared to RNA collected from age matched wild-type whole-heart samples (positive control) and KO heart samples (negative control) ( Figure 1C, IECECs-0 day). In the heart, APJ is primarily expressed by endothelial cells. Therefore, as expected, qRT-PCR detected significantly high level of *Apj* expression in total RNA from isolated endothelial cell population compared to WT whole heart, further confirming that the isolated cell pool contained enriched endothelial cells ( Figure 1C ). As expected, no expression was detected in the germline KO of *Apj* (negative control). Our qRT-PCR analysis revealed robust *Apj* mRNA expression in IECECs, suggesting that it can be used as a faithful coronary endothelial cell culture model to assay for APJ signaling.

Once we verified that the isolated coronary endothelial cells expressed APJ prior to culture, we then subjected these cells to culture to authenticate that the IECECs can be used as an *in vitro* culture model to assay for APJ signaling. To our surprise, qRT-PCR analysis did not show a detectable level of *Apj* expression in 3-day culture of IECECs ( Figure 1C, IECECs - 3 day, n = 4). Our result suggests that *Apj* expression is downregulated when subjected to culture. More importantly, it suggests that *Apj* expression is tissue context-dependent; when the cells are removed from tissue microenvironment, they are likely to progressively lose *Apj* expression. Our result is highly significant in that it provides a caution towards *in vitro* experiments using cell lines because these cell lines might not faithfully present the same transcription profile as they do *in vivo.* Such variation might invalidate the use of cell lines for certain assays. Interestingly, in our IECEC 3-day culture, which show a loss of *Apj* expression, we were able to detect *VegfR2* expression, and its expression was significantly increased when stimulated with its ligand, VEGFA ( Figure 1D ). VEGFR2 is an early marker of endothelial cells, and we predict that IECECs in culture most likely retain their early marker but lose their late and tissue specific marker such as APJ, which is also known to be a venous marker. Collectively, we report that APJ, which is expressed by coronary endothelial cells in embryonic mouse heart and regulate coronary angiogenesis, is downregulated when endothelial cells are removed from their tissue niche and are subjected to culture conditions as the monolayer cells *in vitro* . Therefore, our results provide a caution towards the experimental designs that are reliant on using isolated endothelial cells or cell lines as an *in vitro* model to assay for important biological mechanisms *in vivo* . One of the commonly available endothelial cell lines is Human Umbilical Vein Endothelial Cells (HUVECs). HUVECs are tested for their response to VEGFA as a validation for endothelial cell line and the researchers might mistakenly use this cell line for their assays assuming it to be a faithful endothelial cell line for all purposes. Therefore, supported by our results in this study, we caution that it is important to validate whether cell lines or isolated primary cell cultures maintain similar transcriptomic profile as they do *in vivo* before their use to assay for important biological mechanisms *.*

## Methods

***Isolation of Embryonic Coronary Endothelial Cells***

Embryonic hearts at e14.5 were collected from timed pregnancy. The hearts were homogenized via a series of enzymatic degradations and incubations as per the protocol obtained from Miltenyi Biotec Cardiac Endothelial Cell Isolation Kit protocol (Miltenyi Biotec, Cat # 130-098-373). Cells harvested from the homogenization of embryonic hearts were subjected to a red blood cell lysis solution to avoid clogging of subsequent columns used during the isolation. Following homogenization and red blood cell lysis, primary embryonic coronary endothelial cells were isolated from embryonic mouse hearts according to the manufacturer instructions (Miltenyi Biotec, Cat # 130-098-373). Briefly, the isolation is performed in two-step process. In the first step, the cardiac non-endothelial cells are magnetically labeled and are depleted by separation using magnetic-activated cell sorting (MACS) column, which is placed in the magnetic field of MACS separator. In the second step, the cardiac endothelial cells are magnetically labelled and are separated from cardiac non-endothelial cells by passing through MACS column placed in the magnetic field of a MACS separator. Then the labelled and magnetically separated endothelial cells are collected after removing the column from magnetic field and eluting the magnetically retained cardiac endothelial cells. Subsequently, the cells were collected, centrifuged, and reconstituted in an endothelial growth medium (EGM2-Mv) (Lonza, catalog# CC-4147) for use.

***Detection and quantification of endothelial cells in the isolated cell populations by immunostaining***

To determine the enrichment of endothelial cells (ECs) in an isolated population, we subjected the isolated ECs to 24-hour culture on a cell culture-treated cover glass (CELLTREAT Scientific Products, catalog# 229172) placed in a 24-well tissue culture plate at 70-80% confluency. After about 24 hr culture, the cells were fixed in 4% PFA for 15 minutes in room temperature. After fixation, the cells were washed with 1X PBS and subjected to immunostaining. Primary staining was performed using rabbit anti-ERG 1/2/3 (abcam, catalog# ab92513) antibody to detect endothelial cells and DAPI to detect nuclei of all cells. For primary staining, rabbit anti-ERG 1/2/3 antibody was diluted in 0.5% PBT (1:500 dilution) and the culture was rocked in the primary antibody mix for 24 hr at 4˚C. Following primary staining, the cells were washed with 1X PBS several times and then subjected to secondary staining. The cells were incubated in a solution containing *organism* anti-rabbit Alexa Fluor 555 secondary antibody diluted 1:250 in 0.5% PBT for 4 hours. After secondary antibody staining, the cells were washed several times using 1X PBS solution. After washing, the cells in the round cover glass were mounted onto the slide using few drops of Vectashield Antifade Mounting Medium with DAPI (Vector laboratories, catalog# H-1200-10). A glass coverslip was then placed over the top of the slides and the edges were sealed with clear nail polish. The stained cells in the slides were imaged using a fluorescence microscope to detect coronary endothelial cells in the isolated cell pool.

Images were analyzed and quantified to determine the enrichment of endothelial cells in the isolated cell population. Images were captured from 9 - 10 fields of view (FOVs) following an S-shaped pattern, allowing us to capture images from all representative areas (top, middle, and bottom) containing cells. DAPI + and ERG 1/2/3 + cells were quantified using the Fiji/ImageJ cell counter application.

***qRT-PCR analysis***

Total RNA from either the whole heart sample or the cultured cells were isolated using the Qiagen RNeasy Mini Kit (Fisher Scientific, catalog# 74104) strictly following the manufacturer’s instructions. The total concentration of isolated RNA was measured by using a NanoDrop One device (Fisher Scientific, catalog #13-400-519). The RNA was then subjected gDNA removal using DNase I enzyme mix following the manufacturer’s instruction. cDNA is obtained by performing reverse transcriptase reaction using BIO-RAD iScript reagent as per their recommendation. SYBR green based real time qPCR reaction was prepared using So-Advanced Universal SYBR green Supermix reagents (Bio-Rad, catalog #172-5271) in a 96-well plate and analyzed using the Bio-Rad CFX Opus RT-qPCR equipment. The reactions were run in triplicate for each condition. The expression of each target genes were normalized with GAPDH as a reference gene. The expression data was analyzed using Bio-Rad CFX Opus software.

***Cell culture and experimental treatments***

We subjected the isolated coronary endothelial cells to cell culture at 50-60% confluency. Isolated ECs were cultured in an incubator (37˚C, 5% CO _2_ , 0% O _2_ ) for 24 hours in complete media (EGM 2-Mv, Lonza, catalog# CC-4147). After 24 hours, the ECs were rinsed with 1X PBS under sterile conditions and were subjected to appropriate treatments. Control group was treated with EBM 2 (basal media, Lonza, catalog# 00190860) + 2% FBS (Fisher Scientific, catalog # SH30080.03HI) medium. The experimental groups were treated with 50 ng/mL of VEGFA (Peprotech, catalog# 450-32), 1 μM Elabela (Phoenix Pharmaceuticals, catalog# 007-27), 1 μM Apelin (Phoenix Pharmaceuticals, catalog# 057-18), and 1 μM Elabela + 1 μM Apelin. All the treatments were prepared in EBM 2 + 2% FBS as in control. Each treatment groups were run in triplicate. Cells were cultured in 24 well plates with 500 μL total volume of treatment media. Cells were cultured in treatment media for 48 hours. Cultures were rinsed with 1X PBS each morning and replaced with fresh treatment media. After completion of the treatment period, the wells were rinsed with 1X PBS and processed for subsequent analysis.
